# Impact of Antibiotic Prescribing Patterns on Susceptibilities of Uropathogens in Children below 24 Months Old

**DOI:** 10.3390/antibiotics9120915

**Published:** 2020-12-16

**Authors:** Ji Young Park, Hyun Mi Kang, Eun Min Kwak, Jung-Woo Rhim, Yo Han Ahn, Hyunju Lee, Dae Chul Jeong, Jin Han Kang

**Affiliations:** 1 Department of Pediatrics, Seoul National University Bundang Hospital, Seongnam-si 13620, Gyeonggi-do, Korea; ji8303@gmail.com (J.Y.P.); medicalpooh@daum.net (Y.H.A.); mdopd@hanmail.net (H.L.); 2 Department of Pediatrics, Chung-Ang University Hospital, Seoul 06973, Korea; 3 Department of Pediatrics, Seoul St. Mary’s Hospital, College of Medicine, The Catholic University of Korea, Seoul 06591, Korea; dcjeong@catholic.ac.kr (D.C.J.); kjhan@catholic.ac.kr (J.H.K.); 4 Department of Pediatrics, Samsung Changwon Hospital, College of Medicine, Sung Kyun Kwan University, Changwon-si 51353, Gyeongsangnam-do, Korea; eunmin1007@hanmail.net; 5 Department of Pediatrics, Daejeon St. Mary’s Hospital, College of Medicine, The Catholic University of Korea, Daejeon 34943, Korea; jwrhim@catholic.ac.kr

**Keywords:** urinary tract infections, extended-spectrum β-lactamase, resistance

## Abstract

Monitoring regional antibiotic resistance patterns of uropathogens are important for deciding suitable empirical antibiotics for urinary tract infections (UTIs) in children. This study aimed to investigate regional differences in antimicrobial susceptibility patterns of *E. coli* and *Klebsiella* spp. in children below 24 months old, diagnosed with their first episode of UTI, and to find factors associated with an increased risk for UTI caused by extended-spectrum β-lactamase (ESBL)-producing uropathogens. This was a retrospective cohort study of children diagnosed between 2011 and 2017 in four different hospitals located in four different regions of South Korea; regions A, B, C, and D. The government’s big data repository was used to acquire data on regional antibiotic prescriptions. The pooled antimicrobial susceptibilities of *E. coli* and *Klebsiella* spp. (*n* = 2044) were as follows: ampicillin–sulbactam (61.0%), 3rd generation cephalosporin (3C) (82.8%), and trimethoprim–sulfamethoxazole (72.0%). Multivariate analysis showed that children diagnosed at hospital A (OR, 1.8; 95% confidence interval [CI], 1.2–2.6; *P* = 0.002) and every year that increased in the study period (OR, 1.1; 95% CI, 1.1–1.2; *P* < 0.001) were factors associated with an increased risk for UTIs with ESBL-producers. Regions A and B had significantly higher amounts of oral 3Cs prescribed compared to regions C and D (*P* = 0.009), which correlate with hospitals in the regions that had higher proportions of UTIs with ESBL-producing uropathogens (A and B vs. C and D, *P* < 0.001). Therefore, children in certain regions are at a higher risk for UTIs caused by ESBL-producers compared to other regions, which correlate with regions that had higher amounts of oral 3Cs prescribed.

## 1. Introduction

As one of the most common causes of bacterial infections in childhood, urinary tract infections (UTIs) require prompt diagnosis and initiation of empirical antibiotics to prevent renal scarring [1,2]. The prevalence of UTIs is at the highest in children below 12 months old [3], and this is the age-group in which the kidneys are the most vulnerable to long-term complications warranting the early administration of appropriate antibiotics [4].

Most UTIs are encountered in primary care clinics, and antibiotics are initiated while awaiting culture results. In order to be considered appropriate empirical antibiotics for UTIs, the resistance rate of uropathogens must not exceed 20% [5]. Therefore, monitoring regional antibiotic resistance patterns of uropathogens are extremely important for the decision-making of suitable empirical antibiotics and rendering which antibiotics are ineffective as empirical drugs. Especially in children, it is recommended that clinicians should select initial antibiotics based on sensitivity patterns of the local region [6,7].

Unfortunately, increasing antibiotic resistance is a global problem and threat. Increased exposure to antibiotics plays an important role in the rise in multidrug-resistant (MDR) bacteria through selective pressure [8]. Most of this exposure in children is encountered through oral administration in outpatient clinics; therefore, the increasing amount of antibiotics prescribed in this age group may be the driving force behind the growing proportion of MDR pathogens in bacterial infections [9].

*Escherichia coli* and *Klebsiella* species are the most common causes of UTI in children. Oral antibiotics are as effective as parenteral antibiotics in the treatment of the first episode of UTI in children [10]. However, because of the increase in the proportion of extended-spectrum β-lactamase (ESBL)-producing Enterobacteriaceae, initiating treatment with oral antibiotics is becoming questionable, as ESBLs confer resistance to the commonly used antibiotics, penicillins and cephalosporins, to treat UTI. Treatment options for infections with ESBL-producers in children are limited, especially as fluoroquinolones are not recommended due to safety concerns [11,12].

Other bacteria that cause UTIs in children include *Enterobacter* spp., *Enterococcus* spp., *Proteus* spp., and *Pseudomonas aeruginosa* [13]. *Proteus* spp. are known to promote adhesion and form biofilms that aid in the colonization of the urinary tract and subsequently causing UTIs. Due to its biofilm-forming ability, greater resistance to antibiotics has been reported [14]. Uropathogenic *P. aeruginosa* has many virulence factors such as elastase, phospholipase C, toxin A, exoenzyme S, and biofilm formation, which play an important role in the pathogenesis of UTIs. Despite advances in therapy, *P. aeruginosa* remains a therapeutic challenge in UTIs due to its ability to acquire multiple mechanisms for antibiotic resistance [15].

As MDR and ESBL-producing pathogens are on the rise, and fewer antibiotics are approved for use in children, the clinical and economic impact of emerging antibiotic-resistant bacteria is an important issue [16]. Especially as infections with ESBL-producing pathogens are known to have a worse clinical outcome, the risk factors, treatment options, and infection control measures for infections with ESBL-producing pathogens in children need to be studied further.

The primary aim of this study was to observe regional differences in antimicrobial susceptibility patterns of the two main uropathogens, *E. coli* and *Klebsiella* spp. in children below 24 months old diagnosed with the first episode of UTI. The secondary aim was to investigate the number of antibiotics prescribed in each of the regions to observe the relationship between the amount of antibiotics prescribed and the regions’ increase in UTIs caused by ESBL-producing Enterobacteriaceae.

## 2. Results

### 2.1. Demographics and Etiology

**Figure 1:**
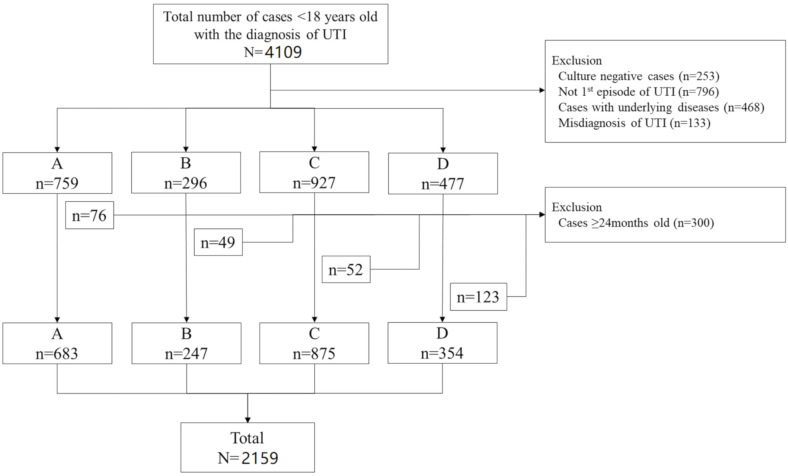
Flow chart of the patients included in this study. A total of 2159 patients below 24 months old were included in the study.

During the 7 year study period between January 2011 to December 2017, 4109 patients below 18 years old were diagnosed with UTIs (Figure 1). Of the 2459 patients below 18 years old that fit the inclusion and exclusion criteria, 87.8% (*N* = 2159) were below 24 months old (Figure 1). A total of 64.7% were male, and the median age of the patients was 4 [interquartile range (IQR) 2.3–7.3] months old. The most prevalent uropathogen was *E. coli* (*n* = 1973, 90.5%), followed by *Klebsiella* spp. (*n* = 71, 3.3%), *Enterobacter* spp. (*n* = 47, 2.2%, and *Enterococcus* spp. (*n* = 33, 1.5%) (Table 1). By hospitals, there was a significant difference in the percentages of the four most common uropathogens; however, in all four hospitals, the most prevalent uropathogen was *E. coli*, followed by *Klebsiella* spp.

### 2.2. Antimicrobial Susceptibility

The antimicrobial susceptibilities of uropathogens *E. coli* and *Klebsiella* spp. (*n* = 2044) were investigated. Susceptibilities to ampicillin, ampicillin–sulbactam, first general cephalosporin, gentamicin, and meropenem were unavailable from hospital C.

**Figure 2:**
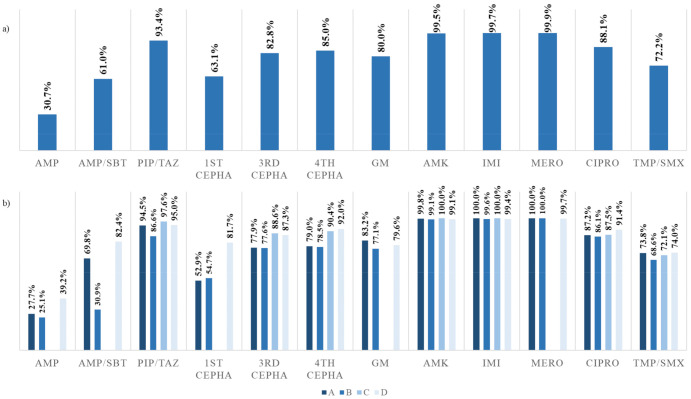
The pooled antimicrobial susceptibilities of (**a**) patients diagnosed with urinary tract infections (UTIs) in all four hospitals, and (**b**) in each of the four hospitals, A to D. AMP, ampicillin; AMP/SBT, ampicillin–sulbactam; PIP/TAZ, piperacillin–tazobactam; 1st CEPHA, 1st generation cephalosporin; 3rd CEPHA, 3rd generation cephalosporin; 4 CEPHA, 4 generation cephalosporin; GM, gentamicin; AMK, amikacin; IMI, imipenem; MERO, meropenem; CIPRO, ciprofloxacin; TMP/SMX, trimethoprim–sulfamethoxazole.

The overall pooled antibiotic susceptibilities from the four hospitals for the following antibiotics were as follows: ampicillin (30.7%), ampicillin–sulbactam (61.0%), piperacillin–tazobactam (93.4%), 3rd generation cephalosporin (3C) (82.8%), 4th generation cephalosporin (85.0%), ciprofloxacin (86.9%), and trimethoprim–sulfamethoxazole (72.0%) (Figure 2a). By hospital, A and B had significantly lower antimicrobial susceptibilities compared to D for ampicillin (*P* < 0.001), ampicillin–sulbactam (*P* < 0.001), and 1st generation cephalosporin (*P* < 0.001). Hospital A and B had significantly lower susceptibilities compared to C and D for 3C (*P* < 0.001) and 4th generation cephalosporin (*P* < 0.001) (Figure 2b).

### 2.3. Multidrug Resistance and ESBL-Producing Enteric Gram-Negative Rods

Overall, the percentage of ESBL-producing *E. coli* and *Klebsiella* spp. was 15.2% (*n* = 310/2044). With the year 2011 as the reference, the odds ratio (OR) of *E. coli* and *Klebsiella* spp. being an ESBL-producer was 2.2 (95% confidence interval [CI], 1.4–3.5; *P* = 0.001) times higher in 2016–2017 (Table 2).

**Figure 3:**
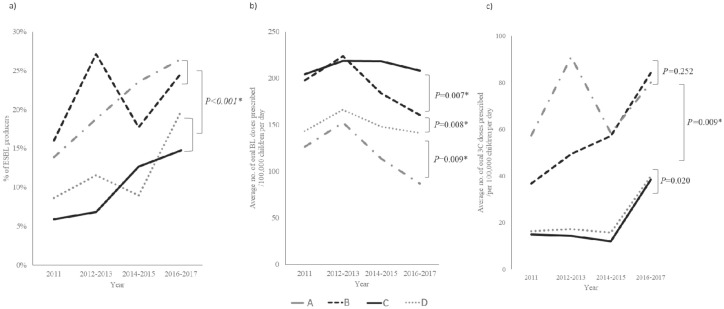
Trends showing (**a**) the proportions of UTIs caused by ESBL-producing uropathogens in hospitals A, B, C, and D. Hospitals A and B had significantly higher proportions of ESBL producers compared to hospitals C and D. (**b**) The average number of oral BL doses prescribed in regions A, B, C, and D. Regions C and B had higher oral BLs prescribed per 100,000 children per day compared to regions D and A. (**c**) The average number of oral 3C doses prescribed in regions A, B, C, and D. Regions A and B had higher oral 3Cs prescribed per 100,000 children per day compared to regions C and D. *P* < 0.05 are shown with an asterisk. BL, β-lactam; ESBL, extended-spectrum β-lactamase; 3C, third-generation cephalosporin.

The overall regional trend in the increase in the proportion of ESBL-producing *E. coli* and *Klebsiella* spp. among uropathogens was investigated. The uropathogens at all four hospitals showed an increasing trend in the proportion of ESBL-producers from 2011 to 2017. However, hospitals A and B had a significantly higher proportion of UTIs in children caused by ESBL-producing uropathogens compared to hospitals C and D (*P* < 0.001) (Figure 3a).

### 2.4. Factors Associated with an Increased Risk for UTIs with ESBL-Producing Pathogens

Factors associated with an increased risk for UTIs with ESBL-producing pathogens were assessed by univariate analyses: sex, age, region, and year diagnosed. Children diagnosed in hospital A (*P* = 0.001), hospital B (*P* = 0.035), as well as the year diagnosed (*P* < 0.001) were shown to be significant. These three factors were included in the final multivariate model, which showed that the OR of children diagnosed in hospital A were 1.8 times higher (95% CI, 1.2–2.6; *P* = 0.002) than that of other hospitals for UTIs with ESBL-producers, and 1.6 times higher (95% CI, 1.0–2.5; *P* = 0.054). In addition, for every year that progressed in the study period, the OR was 1.1 times higher (95% CI, 1.1–1.2; *P* < 0.001) (Table 3).

### 2.5. Total Amount of Antibiotics Prescribed in Each of the Regions That the Four Hospitals Are Located

In all four regions that the four hospitals are located, the average total number of beta-lactam (BL) and 3C doses prescribed in children below 2 years old was 242.8 doses per 100,000 children per day. In region A, the total amount was 247.1 doses per 100,000 children per day; in region B, 274.3 doses; in region C, 254.6 doses; and in region D, 195.2 doses.

For oral BLs, region C had the highest average number prescribed per 100,000 children per day, followed by regions B, D, and A. In all four regions, the average number of oral BL doses prescribed per 100,000 children per day increased up to 2012–2013. Afterward, all four regions showed a decline in the amount prescribed (Figure 3b).

All four regions showed an abrupt increase in the number of oral 3C doses prescribed starting 2014–2015 (Figure 3c). Moreover, regions A and B had significantly higher amounts prescribed compared to regions C and D. This correlates with the hospitals in the regions that had significantly higher proportions of UTIs caused by ESBL-producing uropathogens, hospitals A and B compared to hospitals C and D (*P* < 0.001) (Figure 3a,c).

## 3. Discussion

Many countries are experiencing an increase in antibiotic resistance. Because UTIs are one of the most common causes of bacterial infections in children, and empirical antibiotics need to be prescribed prior to antibiotic tailoring, data on the regional antibiotic resistance patterns of uropathogens are essential for clinical decision-making. This study included 2159 patients below 24 months old, diagnosed with the first episode of UTI from 4 different regions in South Korea, during a 7 year study period from 2011 to 2017. The antimicrobial susceptibilities of *E. coli* and *Klebsiella* spp. were investigated, and factors associated with an increased risk for infection with an ESBL-producing uropathogen were analyzed. Antimicrobial susceptibilities of *E. coli* and *Klebsiella* spp. to ampicillin–sulbactam was 61.0% and 82.8% for 3Cs. Children diagnosed at hospital A and every year that increased during the study period were factors associated with an increased risk for UTIs with ESBL-producers. The regions with significantly higher amounts of oral 3Cs prescribed correlated with the hospitals in the regions that had higher proportions of ESBL-producing uropathogens.

Aminopenicillins are one of the most prescribed antibiotics in outpatient clinics. The pooled prevalence of ampicillin was low—at 30.7%—in this study, with regions A and B below 30%. Similar susceptibilities to ampicillin are seen in adult studies in Korea: one study conducted during 2010–2014 showed 35.3% susceptibility to ampicillin [17], and another study from 2013–2015 showed 30.4% [18]. A meta-analysis on the global prevalence of antibiotic resistance in pediatric UTIs showed similar low susceptibilities, where UTIs caused by *E. coli* showed a pooled prevalence of 46.6% in OECD countries and 20.2% in non-OECD countries [9]. Hence, aminopenicillins should be avoided as empirical treatment options in children with febrile UTI.

Oral antibiotics recommended for the empirical treatment of pediatric UTIs include amoxicillin–clavulanate, trimethoprim–sulfamethoxazole, and 3Cs. Parenteral antibiotics recommended include gentamicin and 3C [19,20]. In our study, the overall pooled susceptibilities to the recommended antibiotics were 61.0% for ampicillin–sulbactam, 72.2% for trimethoprim–sulfamethoxazole, 80.0% for gentamicin, and 82.8% for 3C. This shows that ampicillin–sulbactam and trimethoprim–sulfamethoxazole are unsuitable oral empirical antibiotic choices. Furthermore, assessment by regions showed that the susceptibility rates for 3Cs varied significantly, where regions A and B had susceptibilities below 80% (Figure 2b), making it a debatable choice for empirical antibiotics in those areas.

Globally, there has been an increase in infections caused by ESBL-producing Enterobacteriaceae. A meta-analysis including 16 studies prior to 2014 on the prevalence of ESBL-producing Enterobacteriaceae in pediatric UTIs showed that 14% of pediatric UTIs were caused by ESBL-producing Enterobacteriaceae [21]. Our study period, which goes beyond that, from 2011 to 2017, showed an even higher percentage were 15.1% of *E. coli,* and *Klebsiella* spp. were ESBL-producers. In fact, 10.1% of the *E. coli* and *Klebsiella* spp. were ESBL-producers in 2011; however, a rapid linear increase was observed, reaching 19.0% in 2016–2017 (*P* = 0.001). This has important implications in children because unlike adults who have several oral antibiotic options to choose from for treating infections caused by ESBL-producing Enterobacteriaceae, such as quinolones, there are few alternative oral antibiotic options for children, such as amoxicillin–clavulanate and trimethoprim–sulfamethoxazole. However, the resistance rates to these alternative antibiotics are even higher. Consequently, higher percentages of children with UTIs may experience treatment failure early in the disease course, greater complications such as renal scarring, and prolonged admission periods.

Many studies show that the increasing use of 3Cs is associated with an increased frequency of infections with ESBL-producing Enterobacteriaceae [22,23,24]. Because our study showed that the hospital that the child was diagnosed in was a significant risk factor for infection with ESBL-producing uropathogen, we investigated the amount of oral BLs and 3Cs prescribed in each of the regions that the hospitals were located. In all four regions, a decreasing trend in oral BL prescriptions was observed after 2012–2013. Whereas an increasing trend in the number of oral 3Cs prescribed was noticed beginning 2014–2015 (Figure 3). This depicts the change in antibiotic prescribing behaviors of physicians in outpatient clinics, who are moving towards choosing 3Cs as primary options in the treatment of bacterial infections rather than penicillins. It is difficult to know whether the rise in MDR pathogens comes as a result of this prescribing behavior or the other way around, where the rise in MDR pathogens has been a motivator for the shift in prescribing behaviors. Nevertheless, the rise in the proportion of ESBL-producers in children with UTIs caused by *E. coli* or *Klebsiella* spp. was parallel to the rise in the amount of oral 3C prescribed, regardless of the amount of BLs prescribed. This emphasizes the important role that 3Cs have on the rise in the proportion of ESBL-producers. As the current trend shows a linear increase in the amount of 3Cs prescribed in all four regions, without intervention, a continued increase in the proportion of ESBL-producers causing UTI in children is inevitable.

Enterobacteriaceae are traditionally considered pathobionts, which are potentially pathogenic organisms that normally live as non-harmful microbiota [25]. Continuous exposure to antibiotics causes selective pressure of pathobionts in individuals, leading to the survival and proliferation of MDR bacteria, which subsequently increases the risk of infections caused by these strains. In order to fully understand the effects that oral antibiotics prescribed in outpatient clinics had on children with UTIs, patients with any underlying diseases or predisposing risk factors for carrying ESBL-producing Enterobacteriaceae as normal flora such as recent antibiotic administration, admission, or underlying diseases were excluded, and only patients diagnosed with the first UTI episode were included. Through this study population, it was possible to observe that antibiotic prescribing patterns at a certain region are important when making decisions for empirical antibiotic choices in children with UTIs. Children that lived in areas with higher oral 3C prescription rates had a higher rate of infections with ESBL-producing *E. coli* or *Klebsiella* spp. There are two possible mechanisms for the increase in ESBL-producers in a population with higher oral 3C prescriptions. First, a higher number of children with exposure to 3Cs can lead to a greater percentage of children becoming colonized with ESBL-producing pathobionts as a consequence of direct 3C exposure. Second, it is also possible that children without any direct exposure to 3Cs can acquire ESBL-producing Enterobacteriaceae as normal flora from transmission from other sources, such as contact with other children or family members who are already colonized, or even from the environment [26,27]. In fact, several studies report on the increasing carriage of ESBL-producing gram-negative bacteria in healthy European children, ranging from 2.9–6.7% [28,29,30]. Thus, as more children are directly exposed to 3Cs, and as the number of ESBL-producing Enterobacteriaceae sources increases, the overall percentage of children who have ESBL-producing Enterobacteriaceae in their normal flora will rise.

There were a few limitations to this study. First, antibiotic susceptibilities were based on automated commercial systems for antimicrobial susceptibility testing, which were not the same throughout the four hospitals. However, in clinical practice, each of the automated commercial systems has been reliable and used for clinical decision-making. Second, the total number of antibiotics prescribed was based on the government’s big data system, and certain antibiotics that the government does not screen may not have been included in this study, and the retrospective nature of this multicenter study made it difficult to identify which individuals had direct antibiotics exposure. Finally, data on antibiotic susceptibilities of the uropathogens of each region were collected at a single hospital in each of the regions. Although all the hospitals were tertiary referral hospitals that were representative admission hospitals of each of the regions, a certain amount of selection bias exists. Therefore, caution is warranted when generalizing the results of this study.

## 4. Materials and Methods

### 4.1. Study Population

This was a retrospective cohort study of patients below 24 months of age, who were diagnosed with their first episode of UTI between January 2011 and December 2017 at four referral hospitals located in four different regions of South Korea: hospital A located in the southeast of South Korea (region A), hospital B located in the central region (region B), hospital C located in the northwestern region (region C), and hospital D also located in the northwestern region, but south of hospital C (region D).

Patients who fulfilled all the following criteria were included in this study: (1) no previous history of UTI, (2) ≥38.0 °C detected within 24 h of urinalysis, (3) pyuria (positive leukocytes of ≥10 white blood cells per high-power field on urine microscopy), and (4) positive urine culture (colony count ≥10^5^ of a single organism via catheter sample or >10^5^ via sterile bag collection).

In order to exclude all patients that had may have had predisposing risks factors for being colonized with MDR or ESBL-producing Enterobacteriaceae, the exclusion criteria were applied as follows: patients who had (1) any underlying diseases (hemato-oncologic diseases, gastrointestinal diseases, congenital heart disease, and preterm infants with chronic lung disease) that required recurrent admission, (2) admission history within the previous 6 months, or (3) known predisposing factors for UTIs such as indwelling urinary catheters, neuromuscular diseases, bed-ridden state, and an immunocompromised state.

### 4.2. Study Protocol and Data Collection

The selection of patients to include as study participants were determined by two pediatric infectious disease specialists after a complete review of the electronic medical records. Data acquired included the following: birthdate, sex, admission and discharge date, previous admission histories, urinalysis and culture results, laboratory results, past medical history of underlying diseases, present illness, fever duration, and time of last fever prior to urine culture.

### 4.3. Microbiology and Antimicrobial Susceptibility

Patients with pyuria underwent species identification and antimicrobial susceptibility tests. Three of the four hospitals (hospitals B, C, and D) used MicroScan NegCombo Panel 32 (2006–2008) and Neg Breakpoint Combo Panel 44 (2009–2016) (Siemens Healthcare Diagnostics Inc., West Sacramento, CA, USA), whereas, one hospital (hospital A) used Vitek-2 (bioMérieux, Marcy l’Etoile, France) for species identification and antimicrobial susceptibility tests.

For the investigation of antimicrobial resistance patterns of the two main uropathogens, *E. coli* and *Klebsiella* spp., the Clinical and Laboratory Standards Institute (CLSI) guideline was used to determine the cutoff for antibiotic susceptibilities, and methods suggested in the CLSI guideline was used to confirm the presence of ESBL-producing microorganisms [31]. Antibiotics considered susceptible were categorized as susceptible, whereas antibiotics determined intermediate or resistant were categorized as non-susceptible in the analyses.

### 4.4. Regional Population and Antibiotics Prescribing Patterns in CHILDREN below 24 Months Old

The region was defined by the city in which the hospital was located. In each of the 4 regions included in this study, the population of children below 24 months old each year during 2011–2017 was obtained from the government website, which uses personal identification numbers to count the population (Korean Statistical Information Service, https://kosis.kr). Through the South Korean government’s public big data repository (www.data.go.kr), the total number of parenteral and oral BL including 3C antibiotics prescribed in children below 24 months old was obtained from each of the 4 regions. The criteria “below 24-months old” was chosen because the prevalence of UTI is highest in children below 24 months old, and data can only be retrieved in yearly intervals.

Intravenous antibiotics included all forms of BLs, including penicillins, aminopenicillins, penicillin–penicillinase inhibitors, and cephalosporins. Oral antibiotics that were included were: amoxicillin, amoxicillin–clavulanate, cephradine, cephalexin, cefadroxil, cefaclor, cefuroxime, cefdinir, cefditoren, cefixime, cefpodoxime. Oral antibiotics were classified as either BL (amoxicillin, amoxicillin–clavulanate, cephradine, cephalexin, cefadroxil, cefaclor, cefuroxime) or 3C (cefdinir, cefditoren, cefixime, cefpodoxime). The total number of prescribed doses were analyzed per 100,000 children per day.

### 4.5. Ethical Consideration Statement

The institutional review board (IRB) of all the hospitals involved approved this study (IRB No. Seoul St. Mary’s Hospital KC20RISI0907, Daejeon St. Mary’s Hospital DC17RES10069, Samsung Changwon Hospital 2018-11-004, Seoul National University Bundang Hospital B-1909/562-107). Written informed consent was waived by all hospitals.

### 4.6. Statistical Analysis

All statistical analyses were performed with R version 3.2.1. (R Foundation for statistical computing, Vienna, Austria). Categorical variables were analyzed using the Freeman–Halton extension of the Fisher’s exact probability test, and continuous variables were analyzed using the Kruskal–Wallis H Test. Linear regression analyses were used to investigate the overall temporal trend of ESBL-producers. Univariate logistic regression analyses were used for each of the factors (Sex, age at diagnosis, a region of diagnosis—A, B, C and D, diagnosed year) associated with an increased risk for being infected with an ESBL-producing pathogen, with the binary dependent variable as ESBL-positive or negative. All factors found to be significant (*P* < 0.05) in the univariate analyses were included in the final multivariate model, with each of the factors adjusted for confounding using logistic regression. The chi-squared test for trend in proportions was used to compare trends for the increase in ESBL-producers and antibiotics resistance. The independent two population proportions test was used to compare the difference between the total regional antibiotics use. All the statistical tests performed were 2-tailed, and a *P* value of <0.05 was considered significant.

## 5. Conclusions

To conclude, the susceptibility rates for 3Cs varied by region, making it a debatable choice as empirical antibiotics in certain regions. Children in certain regions are at a higher risk for UTIs caused by ESBL-producers compared to other regions, which correlate with regions that have higher amounts of oral 3Cs prescribed, portraying the important role that 3Cs have on the rise in ESBL-producers. As time progresses, the risk of UTIs caused by ESBL-producers increases; therefore, without intervention, a continued increase in the proportion of ESBL-producers is predicted. Antibiotic prescribing patterns at a certain region should be considered when making decisions for empirical antibiotic choices in children with UTIs. Although this study focused on the impact of oral antibiotic prescriptions, further studies are needed to understand antibiotic resistance gaining profiles depending on the administration routes of antibiotics.

## Figures and Tables

**Table 1:** Demographics of the patients included in this study, the year diagnosed, and uropathogens cultured at each of the hospitals.

	No. of Cases (%)					
	A	B	C	D	Total	P *
	(*N* = 683)	(*N* = 247)	(*N* = 875)	(*N* = 354)	(*N* = 2159)	
Sex, male	457 (66.9)	166 (67.2)	628 (71.8)	254 (71.8)	1505 (69.7)	*0.126*
Median age, months (IQR)	4 (2–6)	4.6 (2.9–6.6)	3 (2–5)	10.8 (7.4–14.8)	4 (2.3–7.3)	*<0.001*
Year of diagnosis						
2011	97 (14.2)	25 (10.1)	115 (13.1)	44 (12.4)	281 (13.0)	*0.424*
2012	64 (9.3)	25 (10.1)	130 (14.9)	53 (15.0)	272 (12.6)	*0.003*
2013	103 (15.1)	28 (11.3)	89 (10.2)	45 (12.7)	265 (12.3)	*0.031*
2014	95 (13.9)	34 (13.8)	117 (13.4)	50 (14.1)	296 (13.7)	*0.984*
2015	113 (16.5)	43 (17.4)	133 (15.2)	56 (15.8)	345 (16.0)	*0.815*
2016	83 (12.2)	50 (20.2)	156 (17.8)	50 (14.1)	339 (15.7)	*0.003*
2017	128 (18.7)	42 (17.0)	135 (15.4)	56 (15.8)	361 (16.7)	*0.352*
Pathogen						
*Escherichia coli*	614 (89.9)	210 (85.0)	813 (92.9)	336 (94.9)	1973 (91.4)	*<0.001*
*Klebsiella* spp.	28 (4.1)	13 (5.3)	27 (3.1)	3 (0.8)	71 (3.3)	*0.011*
*Enterobacter* spp.	25 (3.7)	0	19 (2.2)	3 (0.8)	47 (2.2)	*0.001*
*Enterococcus* spp.	5 (0.7)	14 (5.7)	5 (0.6)	9 (2.5)	33 (1.5)	*<0.001*
*Proteus mirabilis*	3 (0.4)	2 (0.8)	2 (0.2)	0	7 (0.3)	*-*
*Streptococcus* spp.	2 (0.3)	1 (0.4)	2 (0.2)	1 (0.3)	6 (0.3)	*0.973*
*Serratia* spp.	2 (0.3)	2 (0.8)	2 (0.2)	0	6 (0.3)	*-*
*Citrobacter* spp.	0	1 (0.4)	4 (0.5)	0	5 (0.2)	*-*
*Morganella morganii*	2 (0.3)	1 (0.4)	0	0	3 (0.1)	*-*
*Staphylococcus* spp.	1 (0.1)	2 (0.8)	0	0	3 (0.1)	*-*
*Raoultella planticola*	1 (0.1)	0	1 (0.1)	1 (0.3)	3 (0.1)	*-*
*Pseudomonas aeruginosa*	0	0	0	1 (0.3)	1 (0.0)	*-*
*Yersinia enterocolitica*	0	1 (0.4)	0	0	1 (0.0)	*-*

IQR, interquartile range; spp., species. * *P* values represent the statistical difference in the values between the four hospitals.

**Table 2:** The proportion of ESBL-producing uropathogens among children with UTIs caused by *Escherichia coli* and *Klebsiella* spp. by diagnosed year.

Year	Total (*N* = 2044)	ESBL (*N* = 310)	% of ESBL	OR	95% CI	*P*
2011	258	26	10.1	R		
2012–2013	517	61	11.8	1.2	0.7–1.9	*0.475*
2014–2015	595	95	16.0	1.7	1.1–2.7	*0.025*
2016–2017	674	128	19.0	2.2	1.4–3.5	*0.001*

CI, confidence interval; ESBL, extended-spectrum beta-lactamase; OR, odds ratio; UTI, urinary tract infection.

**Table 3:** Multivariate analyses on factors associated with an increased risk for infection with an ESBL-producing uropathogen.

Univariate Analysis					Multivariate Analysis			
	OR	OR (95% CI)		*P*	OR	OR (95% CI)		*P*
		Lower	Upper			Lower	Upper	
Sex	1.0	0.8	1.3	*0.858*				
Age	1.0	1.0	1.0	*0.206*				
Hospital								
A	1.8	1.3	2.7	*0.001*	1.8	1.2	2.6	*0.002*
B	1.6	1.0	2.6	*0.035*	1.6	1.0	2.5	*0.054*
C	0.8	0.6	1.2	*0.304*				
D	R							
Year	1.1	1.1	1.2	*<0.001*	1.1	1.1	1.2	*<0.001*

CI, confidence interval; OR, odds ratio.

## Abbreviations

Extended-spectrum β-lactamase (ESBL); beta-lactam (BL); Clinical and Laboratory Standards Institute (CLSI); interquartile range (IQR); institutional review board (IRB), multidrug resistant (MDR); odds ratio (OR); third generation cephalosporin (3C); urinary tract infections (UTIs).
